# Deciphering the Invdupdel(8p) Genotype–Phenotype Correlation: Our Opinion

**DOI:** 10.3390/brainsci10070451

**Published:** 2020-07-15

**Authors:** Manuela Lo Bianco, Davide Vecchio, Tiziana A. Timpanaro, Alessia Arena, Marina Macchiaiolo, Andrea Bartuli, Laura Sciuto, Santiago Presti, Sarah Sciuto, Annamaria Sapuppo, Agata Fiumara, Lidia Marino, Giulia Messina, Piero Pavone

**Affiliations:** 1 Postgraduate Training Program in Pediatrics, Department of Clinical and Experimental Medicine, University of Catania, 95100 Catania, Italy; laurasciuto93@gmail.com (L.S.); santiagopresti@gmail.com (S.P.); sarah.sciuto@gmail.com (S.S.); annamaria.pan@hotmail.it (A.S.); lidia.m@hotmail.it (L.M.); giuliamessina@hotmail.it (G.M.); 2 Rare Disease and Medical Genetics, Academic Department of Pediatrics, Bambino Gesù Children’s Hospital, 00146 Rome, Italy; davide.vecchio@opbg.net (D.V.); marina.macchiaiolo@opbg.net (M.M.); andrea.bartuli@opbg.net (A.B.); 3 Department of Clinical and Experimental Medicine, University of Catania, 95100 Catania, Italy; timpanarotiziana@yahoo.it (T.A.T.); alessia.arena@gmail.com (A.A.); agatafiumara@yahoo.it (A.F.)

**Keywords:** invdupdel(8p), 8p23.1 sub-band, chromosome 8, genomic rearrangement, inversion, deletion, duplication, CGH-array, FISH

## Abstract

The 8p inverted duplication/deletion is a rare chromosomal rearrangement clinically featuring neurodevelopmental delay, mild to severe cognitive impairment, heart congenital defects and brain abnormalities. Patients affected also present typical facial dysmorphisms and skeletal malformations, and it is thought that the composite clinical picture may fall into the chromosomal rearrangement architecture. With the major aim of better framing its related clinical and diagnostic paths, we describe a patient carrying a de novo invdupde[8p] whose clinical features have not been described so far. Hence, through an extensive genotype–phenotype correlation analysis and by reviewing the dedicated scientific literature, we compared our patient’s features with those reported in other patients, which allows us to place our proband’s expressiveness in an intermediate area, widening the scope of the already known invdupde[8p] genotype–phenotype relationship.

## 1. Introduction

The interstitial inverted duplication of the short arm of chromosome 8, associated with its terminal deletion (invdupdel[8p]), is estimated to affect 1 in 10,000 to 1 in 30,000 newborns [1]. Although few patients have been so far reported, their clinical pictures reveal a homogeneous pattern of features mostly characterized by neurodevelopmental delays/intellectual disabilities, congenital heart defects, agenesis of the corpus callosum and other forms of brain involvement (i.e., atrophy), skeletal malformations, and facial dysmorphisms. Moreover, it has been also reported that the above-mentioned severity of clinical manifestations may strictly depend on the rearrangement size due to the final expression–dosage effect of those genes harbored within [1]. By reviewing the main dedicated scientific literature, we herein describe an 8-year-old girl showing a peculiar phenotype which widens the scope of the already known invdupdel[8p] genotype–phenotype relationship.

## 2. Case Report

The proband was a female admitted in our pediatric unit at the age of 8 years old. The patient is the second-born of non-consanguineous parents, both affected by glaucoma while hypothyroidism was diagnosed in the mother when she was 33 years old. The family history was not informative for any genetic conditions except for a proband’s first cousin affected by Turner syndrome. She was delivered at 39 weeks of gestation (WG) by cesarean section, electively performed due to the known maternal glaucoma, and after a pregnancy complicated by multiple abortion threats and placental abruption mostly occurring during the first trimester and dealt with using isoxsuprine hydrochloride and tranexamic acid. At the birth, her parameters were: weight 2640 g (5 °C), length 46 cm (<3 °C), head circumference 36 cm (95 °C); APGAR score was 9 at 1′, 10:5′. In early infancy her clinical history was characterized by growth delay and the diagnosis of hypothyroidism at the age of 2 months old when she started therapy with levotiroxine, which is still ongoing. Neurodevelopmental milestones were reached with delay; she maintained an erect position at 2 years old, reaching an autonomous deambulation at the age of 3 years old. Up to the age of 4 years old she said only a few words, but the support of logopedic rehabilitation was in the long run ineffective since she currently utters about 10 words and is still not able to elaborate complete meaningful sentences.

At the last physical examination, her parameters were: weight 18.9 kg (<3° pc), height 121 cm (10° pc), head circumference 51 cm (50° pc); skin and annexes were characterized by transient marbling cutaneous reaction with generalized hypertrichosis and signs of a previous sacrococcygeal fistula on the back. While her muscular mass was hypotrophic and hypotonic, lacking in subcutaneous tissue, the skeletal examination showed an extra-rotation of the lower limbs, mainly on the left, varus position of both the knees, flat foot with a pronation tendency, and hypo-eligible osteotendinous reflexes. Moreover, bilateral cutaneous dimples were visible on both elbows and knees, together with a shield chest with inverted nipples and winged shoulder blades. We noted several facial dysmorphisms, as follows: prominent forehead, arched eyebrow, thin nose with rounded tip and anteverse nostrils, flat filter, thin downturned lips, slight micrognathia, and low-set posteriorly rotated ears. Finally, a single palmar crease on the right hand and a bilateral clinodactyly of the IV and V fingers completed her phenotype picture.

**Figure 1:**
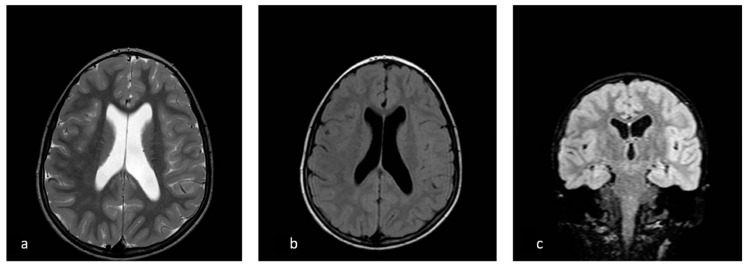
Ventricular asymmetry (left > right) in axial T2 weighted sequence (**a**)**,** axial T2 FLAIR sequence (**b**), and coronal multi planar rendering (**c**)**.**

**Figure 2:**
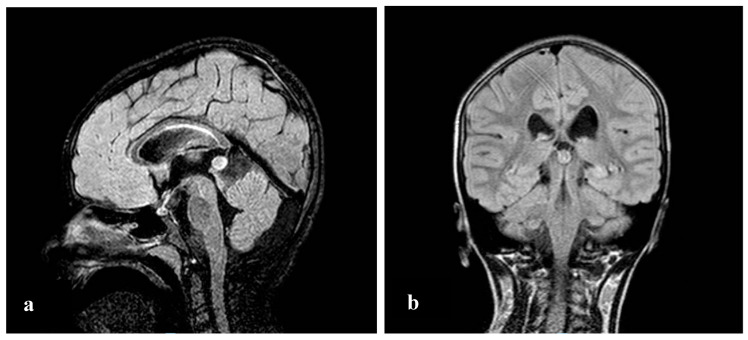
Poly-lobed cystic pineal gland in sagittal (**a**) and coronal (**b**) T2 FLAIR sections.

**Figure 3:**
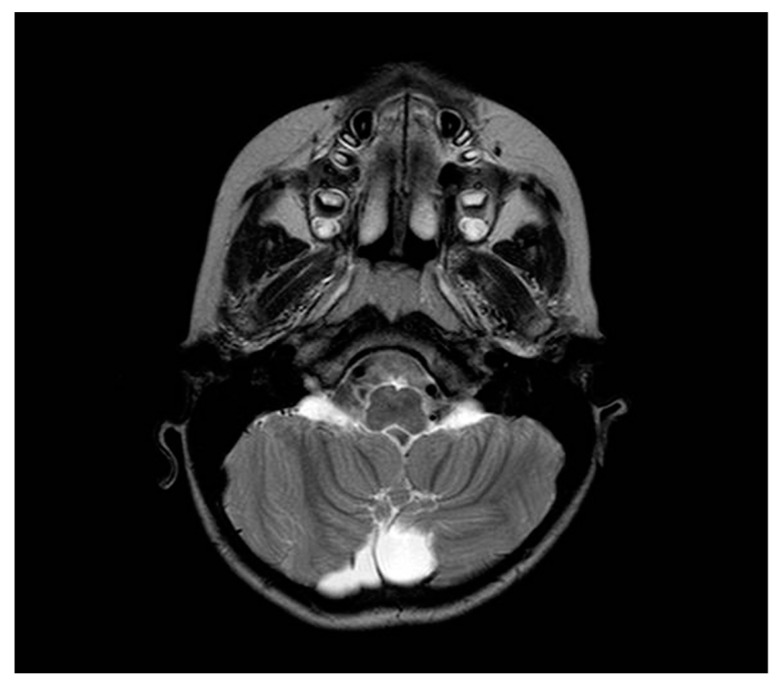
Retrocerebellar cystic ectasia in axial T2 weighted section.

**Figure 4:**
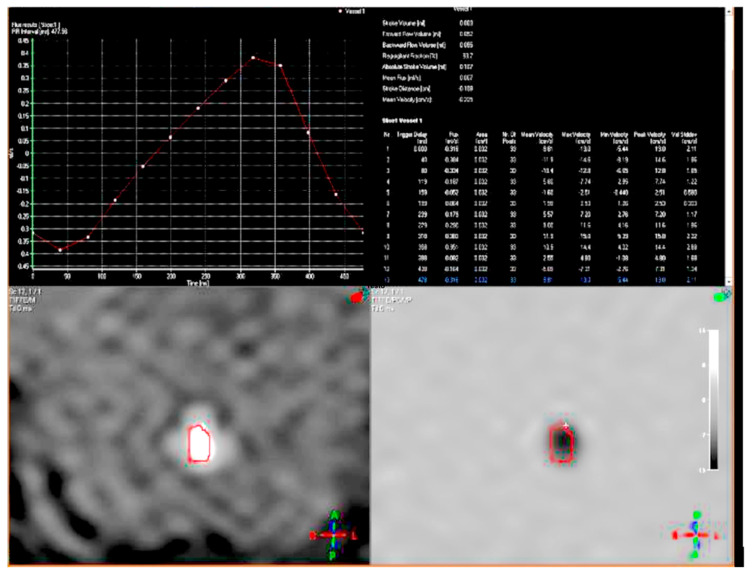
Cine MRI showing a flow turbulence throughout III and IV ventricles.

During the visit, while she had a discrete environmental involvement, she displayed emotiveness and impulsiveness, and a decreased attention span. Blood analyses tested normal except for IgE levels of 522 IU/mL, (range 0–200 UI/mL) and eosinophils 10.5% (range 0.5–5%). Regarding her neurophenotype, the brain Magnetic Resonance Imaging (MRI) performed through T1, T2, and Fluid-Attenuated Inversion Recovery (FLAIR) sequences, revealed a dysmorphic cranial conformation mostly characterized by an incomplete encephalic myelinization, slight dilatation of lateral ventricles with enhancement of liquoral spaces (Figure 1a–c), and a pineal gland’s small ectasia likely due to a partially cystic aspect (Figure 2a,b). The posterior fossa anatomy also showed a moderate cystic cisterna magna’s ectasia (with no bulk-up effect on the subtentorial structures) and a retrocerebellar cystic ectasia (Figure 3). Thus, we also performed a cine MRI which showed a liquor hydrodynamic involvement due to flow turbulence throughout III and IV ventricles (Figure 4). However, since the stroke volume value on the aqueduct of Sylvius was normal, she did not require any peritoneal ventricle derivation. Moreover, no signs of endocranial hypertension were also found at eye examination which instead revealed a pale papilla with clear boundaries and peri-papillar pigmentary ring, in addition to a global chorio-retinic dystrophia. During her follow-up she undertook several electroencephalograms (EEGs), with features that were constantly within the normal range, as were the cardiological examinations performed (echocardiographic examination included). Finally, since at a phoniatric evaluation sialorrhea and extravelic palatin tonsils were noted, she underwent a rhinofibroscopic examination which detected an isolated ogival palate and an audiometric evaluation showing a type C tympanogram with the absence of stapedial reflex on the left.

Human Agilent CGH microarray kit 8 × 60 K showed a complex rearrangement on the short arm of the chromosome 8 characterized by a 6.7 Mb terminal deletion (ranged between 221,611 to 6,914,076 nucleotides) at the 8p23.3p23.1 sub-band in addition to an interstitial inverted duplication spanning the 8p23.1p12 sub-bands of 19.8 Mb that ranged between 12,583,259 to 32,380,292 nucleotides. These rearrangements and their orientations were subsequentially confirmed through Fluorescent in situ hybridization (FISH) analysis by using RP11-45O16 and RP11-139G9 locus-specific probes. Thus, since the proband’s constitutional karyotype was 46,XX,der(8)del(8)(p23.1)invdup(p12p23.1) (Table S1) and her molecular karyotype was arr8p23.2p23 (221,611-6,914,076)×1, 8p23.1p12 (12,583,259-32,380,292)×3, a final diagnosis of invdupdel[8p] syndrome was made.

## 3. Discussion

Inverted duplication deletion of 8p (invdupdel[8p]) is an uncommon chromosome 8 rearrangement with a rated prevalence of 1:10,000–30,000 newborns [1]. Floridia et al. proposed the generation of invdup(8) as a result of an abnormal pairing of chromosomes 8 at the time of maternal meiosis I, followed by an unequal crossover at anaphase I [2]. This mispairing through a subsequential asymmetric breakage of the dicentric chromosome can explain at the same time the observed inverted duplication and its terminal deletion onset [3]. More recently, Giglio et al. demonstrated that the unbalanced cross-over is likely due to the presence of some gene clusters (named ORDRs) spanning the 8p arm which, serving as breakpoints, are thought to be responsible for the generation of different and recurrent chromosome 8p rearrangements, as well as the supernumerary marker chromosome +der(8)(8p23.1pter) and some submicroscopic inversion polymorphisms [4,5]. The counterevidence of this origin comes from the evidence that ORDRs gene clusters are even present on 4p16, where similar inversion polymorphisms have been documented [6,7]. In Table 1 we report the karyotype/CGH-arrays, FISH analysis results and related phenotypes from different cases found in literature compared to our patient.

Regarding to the invdupdel[8p] phenotype, which is usually characterized by facial dysmorphisms and a wide range (to a usually severe degree) of neurodevelopmental delays, several congenital malformations have been described over time as part of its clinical spectrum such as heart defects, facial dysmorphism, skeletal abnormalities and brain anomalies [8,9,10]. By comparing our proband’s features with a 13 patient series reported in the main scientific literature from 2001 to 2018 (Table 1 and Table 2), in this study we review the already known [inv dup del(8p)] genotype–phenotype relationship in order to: (i) better frame the overall clinical picture associated, (ii) highlight some specific features of this rare complex genetic disorder that are helpful in the clinical diagnostic arena, and (iii) improve the knowledge for its management and follow-up. In this view, our proband’s overall clinical presentation falls in a milder phenotype since most of the severe manifestations were not present such as heart congenital defects (HCD) and corpus callosum agenesis (CCa) which are commonly described in these patients. This milder phenotype, compared to other patients, could be determined due a smaller duplication size or a highly suggestive positional effect gained from the inversion [10]. In particular, referring to the heart congenital defects, the most common anomalies reported are patent ductus arteriosus (PDA), ventricular septal defects (VSD) and atrial septal defect (ASD, especially of type II) [1,11,12]. Moreover, while pulmonary stenosis has been described by Hand et al. and by Buysse et al. [13,14], Ergun et al. described a dextrocardia complex case [11] of HCDs, referring to a case of double right ventricle outlet and two tetralogy of Fallot (TOF) cases that were also respectively reported by Gargìa-Santiago et al. [1] and Masuda et al. [12]. Concerning our patient, she only presented a right bundle branch focal block on the ECG. Brain imaging from MRI/TC frequently revealed corpus callosum agenesis (CCA) as a typical anomaly sign, ranging from total to partial agenesis [1,11,12,14,15,16]. However, mild to severe degree brain atrophy is also reported [1,12,17] and a Dandy–Walker variant of the posterior fossa defines some cases [15,17]. Dysmorphic cranial conformation like colpocephaly or flat occiput are also described [15,18]. Moreover, Fan et al. illustrated a case of communicating hydrocephalus and intramedullary cord defects never reported before [15]. In this context, even if our patient’s brain MRI did not show corpus callosum anomalies (which are the main brain defect associated with invdupdel[8p]) or other relevant anomalies, some elements such as a mild ventricular dilatation not related to a significant stroke volume value on cine-MRI sequence integration were highlighted as well as those retrieved in other patients from the presented series analysis (Table 1). Furthermore, regarding the neurobehavioral phenotype, Fisch et al. first described four children with mild to severe cognitive impairment and a significantly lower level of adaptive behavior [19]. By using CARS scores, they also outlined autism or autistic-like features in three out of four children in this series. Three of them also satisfied the DSM-IV-TR diagnosis of Attention Deficit Hyperactivity Disorder (ADHD), showing co-morbid characteristics of hyperactivity and attention deficits [19]. This, in accordance with the above-mentioned literature, can confirm a common neurobehavioral phenotype since our patient was also diagnosed with severe Developmental Delay/Intellectual Disability (DD/ID), ADHD, and speech delay. Among skeletal anomalies, the most frequently represented in literature are kyphoscoliosis, winged shoulders, shield chest, clinodactyly of IV–V fingers and flat feet. A case of syndactyly of II-III fingers was also reported by Knijnenburg et al. [20]. The extra-rotation of lower limbs and varus position of the knees were also remarkable in our patient. In regards to the less frequent abdominal anomalies, we remark on the presence of a case of congenital diaphragmatic hernia [5] and pelvic dysplastic kidneys and hydronephrosis, which have also been reported [15], but these were not detected among our proband’s features. Finally, according to the presented report, we recommend that some ancillary aspects should not be neglected in these patients such as the phoniatric evaluation, since bad occlusion, sialorrhea and hypotonia could affect the severity of the clinical picture. Thus, an audiometric evaluation for the occasional reported neurosensorial hypoacusia [12], and a whole eye examination because of refractory disorders, should be offered both at the diagnosis as well as during the follow-up for the often underrated and occasionally reported ophthalmologic problems such as strabismus [14,18] and/or the chorioretinic dystrophia which was first documented in our patient.

## 4. Conclusions

In conclusion, comprehensive cytogenetic and molecular analyses of the standard karyotype, CGH-array and FISH are mandatory to gain a diagnosis in patients showing a complex phenotype which includes dysmorphic features, neurodevelopmental delay, and major or minor congenital anomalies [21]. In these patients, invdupdel[8p] should be considered in the differential diagnosis [17], as further shown in this study where we highlighted this rare complex genetic disorder’s features whose proper framework may be helpful both on the clinical diagnostic arena as well as to improve patient management and follow-up. Finally, further studies are needed to completely evaluate its genotype–phenotype relationship, especially to widen the scope on the genes–dosage impairment-driven role that we propose once again could be determined by several factors such as breakage sites, genomic size and orientation of the rearrangements involved.

## Figures and Tables

**Table 1:** Karyotype/CGH-arrays, FISH analysis results and phenotypes reported in previous studies and in our patient. +, analysis performed; - analysis not performed; nr, data not reported.

Study	Karyotype/CGH-Arrays	FISH	Phenotype
**Fan et al.**	karyotype of 46,XY,add(8)(p23) 46,XY,der(8)(qter→q24.13::p21.3→p23.3::p23.3→qter)	+	Global developmental delay. Marked hypotonia, weak low cry. Bitemporal low set ears, upslanting palpable fissures, wide nasal bridge, right cleft lip, micrognathia, excess nuchal skin, hypoplastic and widely spaced nipples. Left testis in the inguinal canal. Atrial septal defect, membranous ventricular septal defect, patent ductus arteriosus with a parachute mitral valve. Right pelvic dysplastic kidney and left hydronephrosis. Partial agenesis of the corpus callosum, communicating hydrocephalus, Dandy Walker malformation, intramedullary cord defect.
**Masuda et al.**	der(8) (qter→p23.1::p23.1→p12:)	+	Severe motor delay and mental impairment. Hypotonia. Prominent forehead, posteriorly angulated ears, broad nose with depressed nasal bridge, wide mouth, high-arched palate and downward slanting eyes. Tetralogy of Fallot (TOF). Agenesis of the corpus callosum.
**Vermeesch et al.**	46,XX,del(8)(p23.3) inv dup(8)(p21.1p23.2)/46,XX,del(8)(p21.1)	+	Delayed psychomotor development. Axial hypotonia. Upward slanting palpebral fissures, synophys, and left preauricular tag, low set thumbs with hypotrophic thenars, bilateral clinodactyly of the fifth fingers. Linear areas of depigmentation with bordering areas of hyperpigmentation on the lumbar and presacral region and on both legs. Feeding problems with gastro-esophageal reflux [8].
**Ciccone et al.**	46,XX,psu dic(8)(p23.2)/46,XX,del(8)(p23.1)	+	Severe mental impairment. Asymmetrical face with the left eye lower than the right, left palpebral ptosis, dental malocclusion, zygomatic arch hypoplasia, low set ears, and a short neck with webbing. Kyphoscoliosis, globous abdomen, short upper and lower limbs, premature grey hair.
**Cooke et al.**	46,XX,der(8)dir dup(8)(p21p23.1) del(8)(p23.1p- ter).ish der(8)dir dup(8)(p21p23.1)del(8)(p23.1pter) (wcp8þ,pter -)	+	Global developmental delays. No meaningful speech. Poor auditory attention, impulsiveness and decreased attention span. Upward slanting palpebral fissures, epicanthal folds, low columella with hypoplastic alae nasi, smooth philtrum, thin vermilion to the upper lip, high arched palate, bilateral clinodactyly. Partial complex seizures. Recurrent upper and lower respiratory tract infections. Mild degree of brain atrophy and evidence of a Dandy–Walker variant in the posterior fossa.
**Caglayan et al.**	Del 8p23.1: 6.99 Mb;Dup 8p11.2→8p23.1: 31.51 Mb	nr	Severe cognitive delay. Microcephaly, frontal bossing, malformed ears, thin vermilion of upper lip, abnormal maxilla and mandible, strabismus, coloboma. Corpus callosum agenesis.
**Buysse et al.**	46,XY,der(8)(qter→q24.13::p21.3→p23.3::p23.3→qter) Del 8p23.1l: 6.9 Mb; Dup 8p22: 3.4 Mb;Dup 8qter→24.13: 20.9 Mb	+	Global developmental delay. Hypertelorism, intermittent strabismus of the left eye, hetero-chromia iridis of the right eye, upslanting palpebral fissures, blue sclerae, slight retrognathia, ears posteriorly rotated with a preauricular tag on the left side. Intergluteal hairy dimple. Supravalvular pulmonary stenosis. Bilateral decreased vision with astigmatism and hypermetropia.
**Hand et al.**	Del 8p23.1: 6.8 Mb;Mosaic Del 8p21.2: 1.7Mb; Mosaic Dup p21.2→p23.1:11Mb	-	Cognitive, speech and motor delays. Hypotonia. Bilateral single palmar creases, no clinodactyly. Skin pigmentary abnormalities (faint lines of hyperpigmentation on the backs of the both legs). No evidence of facial dysmorphisms. Cheerful disposition, eager to please.
**Ergun et al.**	Del 8p23.1: 6.71 Mb;Dup 8p11.2!8p23.1: 29.26 Mb	nr	Absent nasal bone and clenched left hand. Enlarged thickened heart walls along with polyvalvular dysplasia. Dilatation of the main pulmonary artery and branches. History of necrotizing enterocolitis. Agenesis of the corpus callosum, enlarged third ventricle and cerebellar hypoplasia.
**Fisch et al.**	arr 8p23.3p23.1(90,616-6,913,476)X1 dn,8p23.1p11.1(12,547,803–43,647,263)X2*3 dn,8p11.23p11.22(39,356,395– 39,505,456)X0 arr 8p23.3p23.1(166,252–6,913,476)X1 dn,8p23.1p11.1(12,547,803–37,028,346)X2*3 dn, 8p11.23p11.22(39,356,395– 39,505,456)X1 arr 8p23.1p21.3(8,117,071–22,366,537)X2*3 dn,8p11 .23p11.22(39,356,395–39,505,456)X0 arr 8p23.3p23.1(166,252–6,913,476)X1 dn,8p23.1p21.3(12,511,655–21,726,774)X2*5 dn, 8p11 .23p11.22(39,356,395– 39,505,456)X0	nr	Long face, wide open eyes. Lack of expressive speech and language. Mild-to-moderate autism. Hyperactivity, restlessness and impulsivity. Large head and prominent forehead. Lethargic, no eye contact. Extremely limited speech/language. Lower than adequate levels of adaptive behavior. Subclinical thought problems and significant anxious/withdrawn behaviors, psychosomatic problems and emotional lability. Severely developmentally and intellectually disabled. Lower than adequate levels of adaptive behavior. Severe autism. Severe thought, withdrawn, social problems. Mild intellectual deficits. Lower than adequate levels of adaptive behavior. ADHD. Thought and social problems.
**Garcìa-Santiago et al.**	Del 8p23.1(330,897–6,420,809): 6.09 Mb; Dup 8p12-- > 8p21 (28,529,348–39,899,187): 11.37 Mb;mosaic dup 8p11.218q11 (41,348,847–48,885,448): 7.54 Mb; dup 8q24.3 (143,626,319–146,157,954): 2.53 Mb Del 8p23.1 (1–6,901,486): 6.90 Mb;Dup 8p12- > 8p23.1 (12,627,630–36,027,465): 23.40 Mb Del 8p23.1 (1–7,233,949): 7.3 Mb;Dup 8p12- > 8p23.1 (12,554,743–34,577,042): 22.03 Mb Del 8p23.1 (1–6,925,869): 6.94 Mb;Dup 8p11.1- > 8p23.1 (12,554,743–41,232,360): 28.76 Mb Del 8p23.1 (1–6,900,000): 6.90 Mb;Dup 8p12- > 8p23.1 (12,296,000–32,800,000): 20.5 Mb Del 8p23.1 (1–6,900,000): 6.90 Mb;Dup 8p11.2- > 8p23.1 (12,296,000–43,700,000): 31 Mb 46,XX,del(8)(p23.3) invdup(8) p21.1p23.2)	+	Mild delayed Speech development. Attention deficit, hyperactivity disorder. Small pointed chin, wide nasal bridge and thick vermilion of the lower lip. Cutis marmorata. Asymmetry of lower limbs. Mild intellectual delay. Hypotonia. Prominent forehead, cupped simple ears, smooth philtrum. Bilateral fifth finger clinodactyly. Fronto-parietal brain atrophy. Moderate intellectual delay. Hypotonia. Protruding tongue in the absence of macroglossia. Patent Ductus Arteriosus (PDA). Brachydactyly. Thinning of the corpus callosum. Severe intellectual delay. Hypotonia. Macrocephaly, narrow and small forehead, facial asymmetry, micrognathia, ptosis, smooth filtrum, macroglossia, spaced teeth, narrow palate, tendency to open mouth and large ears. Corpus callosum agenesis. Valgus feet. Severe intellectual delay. Hypotonia. Brachycephaly, broad forehead with bitemporal narrowing, facial asymmetry, short palpebral fissures, straight and narrow nose, low-set, posteriorly rotated ears, and large mouth. Bilateral fifth fingers clinodactlyly. Ventricular septal defects (VSD). Corpus callosum agenesis. Mild to moderate Intellectual delay. Hypotonia. Protruding ears, straight nose with bulbous tip. High palate, thick vermilion of lips and micrognathia. Double outlet right ventricle (DORV), ventricular septal defects (VSD). Corpus callosum agenesis.
**Knijnenburg et al.**	46 XY	+	Moderate intellectual disability. Flat occiput, epicanthal folds, downturned corners of the mouth, broad based nose, broad hands with tapering fingers and mild 2-3 toe syndactyly. Atrial septal defect. Obesity. Occasionally aggressive outbursts.
**Kumar et al.**	6.7- Mb deletion on chromosome 8p23.3p23.1 and a 31-Mb interstitial duplication on chromosome 8p23.1p11.1.	nr	Global developmental delay. Generalized hypotonia. Broad forehead, low set ears, thick lips, prominent philtrum. Harrison sulcus. History of generalized seizures. Large doubly committed ventricular septal defect (VSD) with left to right shunt and severe hyperkinetic pulmonary artery hypertension. Colpocephaly with complete absence of corpus callosum, prominent ventricles.
**Our patient**	46, XX, der(8)del(8)(p23.1)invdup(p12p23.1)	+	Developmental and speech delay. No meaningful sentences. Hypotonia. Hypothyroidism. Prominent forehead, arched eyebrow, thin nose with rounded tip and anteverse nostrils, flat filter, thin down-turned lips, slight micrognathia, low-set posteriorly rotated ears. Single palmar crease on the right hand and bilateral IV-V fingers clinodactyly. Hypertrichosis, previous sacrococcygeal fistula sign. Extra-rotation of the lower limbs, varus position of both the knees, flat feet. Bilateral cutaneous dimples on both elbows and knees, shield chest, inverted nipples, winged shoulder blades. Emotiveness, impulsiveness, decreased attention span. Dilatation of lateral ventricles, pineal gland’s small ectasia, moderate cystic cisterna magna’s ectasia, retrocerebellar cystic ectasia. Global chorio-retinic dystrophia, pale papilla with clear boundaries, peri-papillar pigmentary ring. Sialorrhea and extravelic palatin tonsils, ogival palate, type C tympanogram with absent stapedial reflex on the left.

**Table 2:** Extensive invdupdel[8p] genotype–phenotype correlation analysis and scientific literature review broken out per article, author, year, number of patients described, patients’ sex, and clinical/instrumental reported anomalies. +, retrieved feature; −, not retrieved feature; nr, not reported.

Article	Year	No. of Patients	Sex	Dysmorphisms	Intellectual Disabilities/Behavioural Disorders	Brain MRI Anomalies	Congenital Heart Defects	Abdominal Anomalies	Skeletal Anomalies
Fan et al.	2001	1	M	+	+	+	+	+	−
Masuda et al.	2002	2	1F/1M	+	+	+	+	−	−
Vermeesch et al.	2003	1	F	+	+	nr	−	−	−
Ciccone et al.	2006	1	F	+	+	nr	−	−	+
Cooke et al.	2008	1	F	+	+	+	−	−	+
Caglayan et al.	2009	1	?	+	+	+	−	−	−
Buysse et al.	2009	1	F	+	+	nr	+	−	−
Hand et al.	2010	1	F	−	+	nr	+	−	−
Ergun et al.	2010	1	F	+	+	+	+	−	−
Fisch et al.	2011	4	2F/2M	+	+	nr	−	−	−
Garcìa−Santiago et al.	2014	7	4F/3M	+	+	+	+	−	+
Knijnenburg et al.	2017	1	M	+	+	nr	+	−	+
Kumar et al.	2018	1	M	+	+	+	+	−	+
Our patient	2020	1	F	+	+	+	−	−	+

## Supplementary Materials

The following are available online at https://www.mdpi.com/2076-3425/10/7/451/s1, Table S1: Full name, OMIM code and function of genes whose loci are harbored within the 8p23.3p23.1 sub-bands, between nucleotidis 221,611 and 6,914,076 of around.

## References

[1] García-Santiago F.A., Martínez-Glez V., Santos F., García-Miñaur S., Mansilla E., Meneses A.G., Rosell J., Granero Á.P., Vallespín E., Fernández L. (2015). Analysis of invdupdel(8p) rearrangement: Clinical, cytogenetic and molecular characterization. Am. J. Med. Genet. A.

[2] Floridia G., Piantanida M., Minelli A., Dellavecchia C., Bonaglia C., Rossi E., Gimelli G., Croci G., Franchi F., Gilgenkrantz S. (1996). The same molecular mechanism at the maternal meiosis I produces mono- and dicentric 8p duplications. Am. J. Hum. Genet..

[3] Ciccone R., Mattina T., Giorda R., Bonaglia M.C., Rocchi M., Pramparo T., Zuffardi O. (2006). Inversion polymorphisms and non-contiguous terminal deletions: The cause and the (unpredicted) effect of our genome architecture. J. Med. Genet..

[4] Giglio S., Broman K.W., Matsumoto N., Calvari V., Gimelli G., Neumann T., Ohashi H., Voullaire L., Larizza D., Giorda R. (2001). Olfactory receptor-gene clusters, genomic-inversion polymorphisms, and common chromosome rearrangements. Am. J. Hum. Genet..

[5] Shimokawa O., Kurosawa K., Ida T., Harada N., Kondoh T., Miyake N., Yoshiura K., Kishino T., Ohta T., Niikawa N. (2004). Molecular characterization of inv dup del(8p): Analysis of five cases. Am. J. Med. Genet. A.

[6] Giglio S., Calvari V., Gregato G., Gimelli G., Camanini S., Giorda R., Ragusa A., Guerneri S., Selicorni A., Stumm M. (2002). Heterozygous Submicroscopic Inversions Involving Olfactory Receptor–Gene Clusters Mediate the Recurrent t(4;8)(p16;p23) Translocation. Am. J. Hum. Genet..

[7] Piccione M., Salzano E., Vecchio D., Ferrara D., Malacarne M., Pierluigi M., Ferrara I., Corsello G. (2015). 4p16.1-p15.31 duplication and 4p terminal deletion in a 3-years old Chinese girl: Array-CGH, genotype-phenotype and neurological characterization. Eur. J. Paediatr. Neurol..

[8] Vermeesch J., Thoelen R., Salden I., Raes M., Matthijs G., Fryns J. (2003). Mosaicism del(8p)/inv dup(8p) in a dysmorphic female infant: A mosaic formed by a meiotic error at the 8p OR gene and an independent terminal deletion event. J. Med. Genet..

[9] de Die-Smulders C.E., Engelen J.J., Schrander-Stumpel C.T., Govaerts L.C., de Vries B., Vles J.S., Wagemans A., Schijns-Fleuren S., Gillessen-Kaesbach G., Fryns J.P. (1995). Inversion duplication of the short arm of chromosome 8: Clinical data on seven patients and review of the literature. Am. J. Med. Genet..

[10] Devriendt K., Matthijs G., Van Dael R., Gewillig M., Eyskens B., Hjalgrim H., Dolmer B., McGaughran J., Bröndum-Nielsen K., Marynen P. (1999). Delineation of the critical deletion region for congenital heart defects, on chromosome 8p23.1. Am. J. Hum. Genet..

[11] Ergün M.A., Kula S., Karaer K., Perçin E.F. (2010). A case with de novo inv dup del(8p) associated with dextrocardia and corpus callosum agenesis. Pediatr. Int..

[12] Masuda K., Nomura Y., Yoshinaga M., Nakamura M., Matsuda Y., Oku S., Miyata K. (2002). Inverted duplication/deletion of the short arm of chromosome 8 in two patients with tetralogy of Fallot. Pediatr. Int..

[13] Hand M., Gray C., Glew G., Tsuchiya K.D. (2010). Mild phenotype in a patient with mosaic del(8p)/inv dup del(8p). Am. J. Med. Genet. A.

[14] Buysse K., Antonacci F., Callewaert B., Loeys B., Fränkel U., Siu V., Mortier G., Speleman F., Menten B. (2009). Unusual 8p inverted duplication deletion with telomere capture from 8q. Eur. J. Med. Genet..

[15] Fan Y.S., Siu V.M. (2001). Molecular cytogenetic characterization of a derivative chromosome 8 with an inverted duplication of 8p21.3-->p23.3 and a rearranged duplication of 8q24.13-->qter. Am. J. Med. Genet..

[16] Kumar V., Roy S., Kumar G. (2018). An Interesting and Unique Case of 8p23.3p23.1 Deletion and 8p23.1p11.1 Interstitial Duplication Syndrome. J. Pediatr. Genet..

[17] Cooke S.L., Northup J.K., Champaige N.L., Zinser W., Edwards P.A.W., Lockhart L.H., Velagaleti G.V.N. (2008). Molecular cytogenetic characterization of a unique and complex de novo 8p rearrangement. Am. J. Med. Genet. Part A.

[18] Caglayan A.O., Engelen J.J.M., Ghesquiere S., Alofs M., Saatci C., Dundar M. (2009). Fluorescence in situ hybridization and single nucleotide polymorphism of a new case with inv dup del(8p). Genet. Couns..

[19] Fisch G.S., Davis R., Youngblom J., Gregg J. (2011). Genotype-phenotype association studies of chromosome 8p inverted duplication deletion syndrome. Behav. Genet..

[20] Knijnenburg J., Uytdewilligen M.E.W., van Hassel D.A.C.M., Oostenbrink R., Eussen B.H.J., de Klein A., Brooks A.S., van Zutven L.J.C.M. (2017). Postzygotic telomere capture causes segmental UPD, duplication and deletion of chromosome 8p in a patient with intellectual disability and obesity. Eur. J. Med. Genet..

[21] Piro E., Consiglio V., Agrifoglio M., Sireci F., Ballacchino A., Salvago P., Martines F., Graziano F., Busè M., Sanfilippo C. (2013). Diagnosis and follow-up of complex congenital malformations/mental retardation (MRA/MR). Acta Med. Mediterr..

