# Supplementary material for: Deciphering the Invdupdel(8p) Genotype–Phenotype Correlation: Our Opinion
Source: Brain Sci. 2020 Jul 15;10(7):451. doi: 10.3390/brainsci10070451 (PMC7408450; doi:10.3390/brainsci10070451)
Supplement: Supplementary file 1 [file brainsci-10-00451-s001.pdf]

Table S1. Full name, OMIM code and function of genes whose loci are harbored within the 8p23.3p23.1 sub-bands, between nucleotidis 221,611 and 6,914,076 of around.

GENE	Full Name	OMIM	Function
AGPAT5	1-acylglycerol-3-phosphate O-acyltransferase 5	614796	AGPAT5 is a member of a family of 1-acyl-sn-glycerol 3-phosphate acyltransferases (EC 2.3.1.51), also known as lysophosphatidic acid acyltransferases, that catalyze the acylation of lysophosphatidic acid to phosphatidic acid, the precursor of all glycerolipids (summary by Lu et al., 2005).[20]
ANGPT2	Angiopoietin 2	601922	Binds to TEK/TIE2, competing for the ANGPT1 binding site, and modulating ANGPT1 signaling. Can induce tyrosine phosphorylation of TEK/TIE2 in the absence of ANGPT1. In the absence of angiogenic inducers, such as VEGF, ANGPT2-mediated loosening of cell-matrix contacts may induce endothelial cell apoptosis with consequent vascular regression. In concert with VEGF, it may facilitate endothelial cell migration and proliferation, thus serving as a permissive angiogenic signal. Show less *Source: UniProt*
ARHGEF10	Rho guanine nucleotide exchange factor 10	608136	May play a role in developmental myelination of peripheral nerves. *Source: UniProt* ARHGEF10 is a member of the family of Rho guanine nucleotide exchange factors (GEFs), which are implicated in neural morphogenesis and connectivity and regulate the activity of small Rho GTPases by catalyzing the exchange of bound GDP by GTP (Verhoeven et al., 2003). [21]
CLN8	CLN8 transmembrane ER and ERGIC protein	607837	Could play a role in cell proliferation during neuronal differentiation and in protection against cell death. *Source: UniProt*
CSMD1	CUB and Sushi multiple domains 1	608397	Potential suppressor of squamous cell carcinomas. *Source: UniProt* Kraus et al. (2006) found that rat Csmd1 blocked classical complement pathway activation in a manner comparable to rat Crry, but it did not block alternative pathway activation. In situ hybridization and neuron immunolabeling showed that Csmd1 was synthesized in the developing central nervous system and in epithelial tissues, with particular enrichment in the nerve growth cone.[22]
DEFA1	Defensin alpha 1	125220	Defensin 1 and defensin 2 have antibacterial, fungicide and antiviral activities. Has antimicrobial activity against Gram-negative and Gram-positive bacteria. Defensins are thought to kill microbes by permeabilizing their plasma membrane. *Source: UniProt*
DEFA3	Defensin alpha 3	604522	Defensin 2 and defensin 3 have antibiotic, fungicide and antiviral activities. Has antimicrobial activity against Gram-negative and Gram-positive bacteria.

			Defensins are thought to kill microbes by permeabilizing their plasma membrane. *Source: UniProt*
DEFA4	Defensin alpha 4	601157	Has antimicrobial activity against Gram-negative bacteria, and to a lesser extent also against Gram-positive bacteria and fungi. Protects blood cells against infection with HIV-1 (in vitro). Inhibits corticotropin (ACTH)-stimulated corticosterone production. *Source: UniProt*
DEFA5	Defensin alpha 5	600472	Has antimicrobial activity against Gram-negative and Gram-positive bacteria. Defensins are thought to kill microbes by permeabilizing their plasma membrane. All DEFA5 peptides exert antimicrobial activities, but their potency is affected by peptide processing. *Source: UniProt*
DEFA6	Defensin alpha 6	600471	Has very low antimicrobial activity against Gram-negative and Gram-positive bacteria. May protect cells against infection with HIV-1. *Source: UniProt*
DEFB1	Defensin beta 1	602056	Has bactericidal activity. May act as a ligand for C-C chemokine receptor CCR6. Positively regulates the sperm motility and bactericidal activity in a CCR6-dependent manner. Binds to CCR6 and triggers Ca2+ mobilization in the sperm which is important for its motility (PubMed…Show more *Source: UniProt*
DLGAP2	DLG associated protein 2	605438	May play a role in the molecular organization of synapses and neuronal cell signaling. Could be an adapter protein linking ion channel to the subsynaptic cytoskeleton. May induce enrichment of PSD-95/SAP90 at the plasma membrane. *Source: UniProt*
ERICH1-AS1	DLG associated protein 2	605438	The DLGAP family of proteins, including DLGAP2, interact with the PSD-95/SAP90 protein (DLG4; 602887), which in turn interacts with Shaker-type potassium channels (see 176260) and other molecules and clusters with these at synaptic junctions [23]
FBXO25	f-box protein 25	609098	Substrate-recognition component of the SCF (SKP1-CUL1-F-box protein)-type E3 ubiquitin ligase complex. May play a role in accumulation of expanded polyglutamine (polyQ) protein huntingtin (HTT) (By similarity). *Source: UniProt*
MCPH1	Microcephalin 1	607117	Implicated in chromosome condensation and DNA damage induced cellular responses. May play a role in neurogenesis and regulation of the size of the cerebral cortex. *Source: UniProt*
MYOM2	Myomesin 2	603509	Major component of the vertebrate myofibrillar M band. Binds myosin, titin, and light meromyosin. This binding is dose dependent. *Source: UniProt*
